# Activity of ceftolozane/tazobactam and imipenem/relebactam against Gram-negative clinical isolates collected in Mexico—SMART 2017–2021

**DOI:** 10.1093/jacamr/dlae077

**Published:** 2024-05-24

**Authors:** James A Karlowsky, Sibylle H Lob, Fakhar Siddiqui, Thales Polis, Jose L Vallejo, Katherine Young, Mary R Motyl, Daniel F Sahm

**Affiliations:** IHMA, 2122 Palmer Drive, Schaumburg, IL 60173, USA; Department of Medical Microbiology and Infectious Diseases, Max Rady College of Medicine, Room 543—745 Bannatyne Avenue, University of Manitoba, Winnipeg, MB R3E 0J9, Canada; IHMA, 2122 Palmer Drive, Schaumburg, IL 60173, USA; Merck & Co., Inc., 126 East Lincoln Avenue, Rahway, NJ 07065, USA; MSD Brasil, Av. Chucri Zaidan, 296—Vila Cordeiro, São Paulo 04583-110, Brazil; MSD Mexico, Av. San Jerónimo 369, Tizapán San Ángel, Tizapán, Álvaro Obregón, 01090 Mexico City, Mexico; Merck & Co., Inc., 126 East Lincoln Avenue, Rahway, NJ 07065, USA; Merck & Co., Inc., 126 East Lincoln Avenue, Rahway, NJ 07065, USA; IHMA, 2122 Palmer Drive, Schaumburg, IL 60173, USA

## Abstract

**Objectives:**

To investigate the activities of ceftolozane/tazobactam and imipenem/relebactam against *Escherichia coli*, *Klebsiella pneumoniae* and *Pseudomonas aeruginosa* isolated from hospitalized patients in Mexico in 2017–2021.

**Methods:**

MICs were determined by CLSI broth microdilution and interpreted using CLSI M100 breakpoints. β-Lactamase genes were identified in ceftolozane/tazobactam-, imipenem/relebactam-, and/or imipenem-non-susceptible isolates.

**Results:**

Ceftolozane/tazobactam and imipenem/relebactam inhibited 89% and 99% of *E. coli* isolates (*n *= 2337), and 87% and 94% of *K. pneumoniae* isolates (*n *= 1127). Sixty-four percent of *E. coli* and 47% of *K. pneumoniae* had an ESBL non-carbapenem-resistant Enterobacterales (ESBL non-CRE) phenotype. Eighty-six percent and 91% of ESBL non-CRE *E. coli* and *K. pneumoniae* were ceftolozane/tazobactam susceptible, and 99.9% and 99.8% were imipenem/relebactam susceptible. Ceftolozane/tazobactam was the most active agent studied against *P. aeruginosa* (*n *= 1068; 83% susceptible), 9–28 percentage points higher than carbapenems and comparator β-lactams excluding imipenem/relebactam (78% susceptible). Ceftolozane/tazobactam remained active against 35%–58%, and imipenem/relebactam against 32%–42%, of *P. aeruginosa* in meropenem-, piperacillin/tazobactam-, and cefepime-non-susceptible subsets. The majority of isolates of ceftolozane/tazobactam-non-susceptible *E. coli* carried an ESBL, whereas among ceftolozane/tazobactam-non-susceptible *K. pneumoniae* and *P. aeruginosa*, the majority carried carbapenemases. The most prevalent carbapenemase observed among *E. coli* (estimated at 0.7% of all isolates), *K. pneumoniae* (4.8%) and *P. aeruginosa* (10.0%) was an MBL. Almost all imipenem/relebactam-non-susceptible *E. coli* and *K. pneumoniae* carried MBL or OXA-48-like carbapenemases, whereas among imipenem/relebactam-non-susceptible *P. aeruginosa*, 56% carried MBL or GES carbapenemases.

**Conclusions:**

Ceftolozane/tazobactam and imipenem/relebactam may provide treatment options for patients infected with β-lactam-non-susceptible Gram-negative bacilli, excluding isolates carrying an MBL- or OXA-48-like carbapenemase.

## Introduction

ESBL-producing and carbapenem-resistant Enterobacterales (CRE), and carbapenem-resistant *Pseudomonas aeruginosa* are frequently MDR and leave clinicians with few or no safe and effective treatment choices. Current surveillance data for both newer and established antimicrobial agents across different geographies inform empirical treatment decisions in those locations as well as supporting global resistance monitoring and drug development efforts.^1–3^

Ceftolozane/tazobactam, an antipseudomonal cephalosporin combined with a longstanding β-lactamase inhibitor, is a newer antimicrobial agent approved in Mexico for the treatment of complicated urinary tract and intra-abdominal infections, and hospital-acquired and ventilator-associated bacterial pneumonia (HAP/VAP).^4^ Imipenem/relebactam is a combination of imipenem/cilastatin (carbapenem/renal dehydropeptidase inhibitor) with relebactam, a non-β-lactam diazabicyclooctane inhibitor of class A and C β-lactamases,^5,6^ that is approved by the US FDA for the treatment of complicated urinary tract and intra-abdominal infections, and HAP/VAP,^7^ and by the EMA for the treatment of infections due to aerobic Gram-negative organisms in adults with limited treatment options; for the treatment of HAP, including VAP, in adults; and for the treatment of bacteraemia that occurs in association with, or is suspected to be associated with, HAP or VAP, in adults.^8^ Imipenem/relebactam is not approved currently for clinical use in Mexico.

Published surveillance data for ceftolozane/tazobactam and imipenem/relebactam tested against clinical isolates of Enterobacterales and *P. aeruginosa* from Mexico are very limited.^9,10^ Therefore, in the current study, we evaluated the activity of ceftolozane/tazobactam, imipenem/relebactam, and nine comparator agents against common Gram-negative pathogens (*Escherichia coli*, *Klebsiella pneumoniae*, *P. aeruginosa*) collected in Mexico by the SMART (Study for Monitoring Antimicrobial Resistance Trends) global surveillance programme and also identified β-lactamase resistance mechanisms in phenotypically resistant isolate subsets.

## Materials and methods

### Bacterial isolates

From 2017 to 2021, eight clinical laboratory sites in Mexico participated in the SMART global surveillance programme; six of the eight sites participated in all five years, one additional site participated in 2019, and one additional site participated in 2021. Sites were each asked to collect consecutive, clinically significant isolates of aerobic or facultatively anaerobic Gram-negative bacilli from intra-abdominal infection (IAI; 75 isolates in 2017 and 50 isolates/year in 2018–2021), lower respiratory tract infection (LRTI; 100 isolates/year), urinary tract infection (UTI; 75 isolates in 2017 and 50 isolates/year in 2018–2021), and bloodstream infection (BSI; 50 isolates/year in 2018–2021 only) samples. Isolates were restricted to one isolate per patient per Gram-negative species per year. Organism-specific quotas are not used in the collection of isolates by the SMART global surveillance programme. All isolates were shipped to IHMA (Schaumburg, IL, USA) where organism identity was confirmed using MALDI-TOF MS (Bruker Daltonics, Billerica, MA, USA), and antimicrobial susceptibility and molecular testing was performed.

### Antimicrobial susceptibility testing

MICs were determined by the CLSI reference broth microdilution method^11^ on custom-made dehydrated broth microdilution panels manufactured by TREK Diagnostic Systems (Thermo Fisher Scientific, Oakwood Village, OH, USA) in 2017 and frozen broth microdilution panels prepared at IHMA in 2018–2021. MICs were interpreted using 2023 CLSI M100 breakpoints.^12^ An ESBL non-CRE phenotype was defined for *E. coli* and *K. pneumoniae* as an isolate testing as non-susceptible to ceftriaxone (MIC ≥2 mg/L) and susceptible to ertapenem (MIC ≤0.5 mg/L). For *E. coli* and *K. pneumoniae*, the EUCAST breakpoint for amikacin^13^ was used to interpret MIC data for that agent because the concentration range tested did not extend low enough to include the revised 2023 CLSI susceptible breakpoint for that agent.^12^

### Screening for β-lactamase genes

Isolates that were ceftolozane/tazobactam-, imipenem/relebactam-, and/or imipenem-non-susceptible were screened for genes encoding β-lactamase genes by short-read WGS (ResFinder database; only *P. aeruginosa* isolates collected in 2020 and 2021) or by PCR and Sanger sequencing, as described previously.^14–16^ All isolates from 2017 to 2019 that met the screening criteria were tested. Isolates characterized in 2020 and 2021 were a random sample of those that met the screening criteria. In total, 274 of 287 (95.5%) Enterobacterales isolates that qualified for molecular characterization in 2020 and 2021 were characterized, as were 185 of 238 (77.7%) qualified *P. aeruginosa* isolates. The percentage of characterized, qualifying isolates collected in 2020 and 2021 was factored into the estimation of carbapenemase rates.

## Results

A total of 4880 isolates of Enterobacterales and 1068 isolates of *P. aeruginosa* were collected by the eight laboratories from 2017 to 2021 (Table S1, available as Supplementary data at *JAC-AMR* Online). *E. coli* (*n *= 2337) and *K. pneumoniae* (*n *= 1127) were the two most common species of Gram-negative bacilli isolated and accounted for 71% of the 4880 isolates of Enterobacterales. Together, *E. coli*, *K. pneumoniae* and *P. aeruginosa* (the third most frequently isolated species of Gram-negative bacilli) accounted for 67% of all Gram-negative bacilli collected by the SMART global surveillance programme in Mexico from 2017 to 2021. Among *E. coli*, *K. pneumoniae* and *P. aeruginosa* isolates, 13.5%, 37.3% and 51.6%, respectively, were collected from patients with LRTI; 35.5%, 26.0% and 17.7% from patients with UTI; 33.5%, 16.8% and 17.6% from patients with IAI; and 17.5%, 19.9% and 12.7% from patients with BSI (Table S1). An infection source was not identified for 0.1% of *E. coli*, 0.1% of *K. pneumoniae* and 0.4% of *P. aeruginosa* isolates.

Ceftolozane/tazobactam and imipenem/relebactam were active against 89% and 99% of *E. coli* isolates, respectively, and against 87% and 94% of *K. pneumoniae* isolates (Table 1). Imipenem, meropenem and ertapenem percent susceptible values ranged from 97% to 99% for *E. coli* and from 91% to 93% for *K. pneumoniae*. The addition of relebactam to imipenem had only a minor impact on percent susceptible values for both *E. coli* (0.5%) and *K. pneumoniae* (1.4%).

**Table 1.** Antimicrobial susceptibility of all clinical isolates of *E. coli*, *K. pneumoniae* and *P. aeruginosa* and resistant phenotype subsets collected by the SMART global surveillance programme in Mexico from 2017 to 2021

Species		% Susceptible										
Resistant phenotype	*n*	C/T	IPM/REL	IMI	MEM	ETP	FEP	CAZ	CRO	TZP	LVX^a^	AMK^b^
*E. coli*	2337	89.3	99.0	98.5	98.8	97.0	38.4	42.4	33.1	74.1	30.1	90.8
ESBL non-CRE^c^	1500	86.4	99.9	99.7	100	100	8.3	14.2	0	67.8	13.7	88.3
*K. pneumoniae*	1127	87.1	93.6	92.2	92.5	90.6	47.6	48.4	44.3	64.0	55.2	90.4
ESBL non-CRE^c^	526	91.4	99.8	99.4	99.8	100	7.0	9.5	0	47.5	30.2	89.4
*P. aeruginosa*	1068	83.1	77.8	55.1	62.1	NA	73.9	71.9	NA	69.0	65.3	80.1
MEM-non-susceptible	405	57.5	42.0	2.0	0	NA	43.2	41.7	NA	35.6	31.9	51.4
TZP-non-susceptible	331	47.4	41.4	19.6	21.1	NA	20.2	16.0	NA	0	26.3	48.3
FEP-non-susceptible	279	35.1	31.5	16.1	17.6	NA	0	5.7	NA	5.4	19.7	39.1

AMK, amikacin; CAZ, ceftazidime; CRE, carbapenem-resistant Enterobacterales; CRO, ceftriaxone; C/T, ceftolozane/tazobactam; ETP, ertapenem; FEP, cefepime; IPM, imipenem; IPM/REL, imipenem/relebactam; LVX, levofloxacin; MEM, meropenem; NA, not applicable; TZP, piperacillin/tazobactam.

^a^Levofloxacin was only tested against Enterobacterales in 2018–2021.

^b^For *E. coli* and *K. pneumoniae*, the EUCAST breakpoint for amikacin was used because the concentration range tested did not extend low enough to include the revised 2023 CLSI susceptible breakpoint for that agent.

^c^ESBL non-CRE phenotype was defined by an isolate testing non-susceptible to ceftriaxone (MIC ≥2 mg/L) and susceptible to ertapenem (MIC ≤0.5 mg/L).

Among *E. coli* and *K. pneumoniae*, 64% and 47% of isolates, respectively, had an ESBL non-CRE phenotype resulting in overall percent susceptible values of <50% for cefepime, ceftazidime and ceftriaxone. Only 61% of all *K. pneumoniae* isolates, 51% of all *E. coli* isolates, 34% of ESBL non-CRE *K. pneumoniae* isolates, and 27% of ESBL non-CRE *E. coli* isolates had a cefepime-susceptible or cefepime susceptible-dose dependent phenotype. Percent susceptible values for ESBL non-CRE phenotype *E. coli* and *K. pneumoniae* were 86% and 91% for ceftolozane/tazobactam and 99.9% and 99.8% for imipenem/relebactam. Only 68% of ESBL non-CRE phenotype *E. coli* and 48% of ESBL non-CRE phenotype *K. pneumoniae* were piperacillin/tazobactam-susceptible. Thirty percent or less of ESBL non-CRE phenotype *E. coli* and *K. pneumoniae* were levofloxacin-susceptible. As expected, the addition of relebactam to imipenem had only a minor impact on the percent susceptible value (0.4% or less) for ESBL non-CRE phenotype isolates of *E. coli* and *K. pneumoniae* as susceptibilities to imipenem alone were ≥99.4%. Percent susceptible values for ceftolozane/tazobactam ranged from 85% (LRTI) to 90% (BSI, IAI and UTI) among *E. coli* specimen sources and from 83% (BSI) to 91% (IAI) among *K. pneumoniae* specimen sources (Table S2). Percent susceptible values for imipenem/relebactam had narrower ranges than ceftolozane/tazobactam, from 98% (LRTI) to >99% (IAI and UTI) for *E. coli* specimen sources, and from 92% (BSI) to 95% (IAI) for *K. pneumoniae* specimen sources. Amikacin percent susceptible values for ESBL non-CRE phenotype *E. coli* and *K. pneumoniae* were ∼10% lower than for imipenem/relebactam, imipenem, meropenem and ertapenem.

Ceftolozane/tazobactam was the most active of the studied agents against *P. aeruginosa*, inhibiting 83% of collected isolates, 9–28 percentage points higher than the comparator β-lactams (Table 1). Susceptibility of *P. aeruginosa* to imipenem/relebactam was 78%, 23 percentage points higher than imipenem alone. Ceftolozane/tazobactam and imipenem/relebactam remained active against 35%–58% and 32%–42% of *P. aeruginosa* isolates with meropenem-non-susceptible, piperacillin/tazobactam-non-susceptible, and cefepime-non-susceptible phenotypes. Percent susceptible values for ceftolozane/tazobactam ranged from 71% (UTI) to 89% (LRTI) among *P. aeruginosa* specimen sources compared with a range of percent susceptible values of 66% (UTI) to 82% (LRTI) for imipenem/relebactam (Table S2).

Carbapenemases were relatively rare among clinical isolates of *E. coli* in Mexico (estimated at 1.3%) but were more common among *K. pneumoniae* (7.5%) and *P. aeruginosa* (13.0%) (Table 2). MBL was the most prevalent carbapenemase type, observed among *E. coli* (0.7% of all isolates), *K. pneumoniae* (4.8%) and *P. aeruginosa* (10.0%) isolates. Molecular characterization of ceftolozane/tazobactam-non-susceptible *E. coli* showed that 96% (234/243) of characterized isolates carried an ESBL, with 77% (187/243) of isolates carrying only an ESBL and no other β-lactamase (Table 3). In comparison, among ceftolozane/tazobactam-non-susceptible *K. pneumoniae* and *P. aeruginosa*, the majority of isolates carried carbapenemases, predominantly MBLs, with a smaller number carrying OXA-48-like (*K. pneumoniae*) or GES (*P. aeruginosa*) enzymes. Among imipenem/relebactam-non-susceptible *E. coli* and *K. pneumoniae*, almost all isolates carried MBL or OXA-48-like carbapenemases, whereas among imipenem/relebactam-non-susceptible *P. aeruginosa*, 56% (118/212) of characterized isolates carried an MBL or GES carbapenemase. KPC (only identified in isolates of ceftolozane/tazobactam-non-susceptible *K. pneumoniae*), OXA-48-like (identified in ceftolozane/tazobactam-non-susceptible and imipenem/relebactam-non-susceptible *E. coli* and *K. pneumoniae*), and GES (identified in ceftolozane/tazobactam-non-susceptible and imipenem/relebactam-non-susceptible *K. pneumoniae* and *P. aeruginosa*) were rarely identified in isolates from Mexico.

**Table 2.** Estimated carbapenemase rates among *E. coli*, *K. pneumoniae* and *P. aeruginosa* isolates collected by the SMART global surveillance programme in Mexico from 2017 to 2021

	Pathogen (total number of isolates) Estimated percentage of total number of isolates with carbapenemase		
Carbapenemase	*E. coli* (*n* = 2337)	*K. pneumoniae* (*n* = 1127)	*P. aeruginosa* (*n* = 1068)
MBL	0.7	4.8	10.0
KPC	0	0.6	0
OXA-48-like	0.6	2.0	0
GES	0	0.1	3.0

GES, Guiana ESBL; KPC, *Klebsiella pneumoniae* carbapenemase; OXA, oxacillinase.

**Table 3.** Acquired β-lactamases detected in ceftolozane/tazobactam-non-susceptible and imipenem/relebactam-non-susceptible *E. coli*, *K. pneumoniae* and *P. aeruginosa*^a^ collected in Mexico by SMART from 2017 to 2021

	Ceftolozane/tazobactam-non-susceptible			Imipenem/relebactam-non-susceptible		
Genotype	*E. coli*	*K. pneumoniae*	*P. aeruginosa*	*E. coli*	*K. pneumoniae*	*P. aeruginosa*
	(*n* = 251)	(*n* = 145)	(*n* = 181)	(*n* = 23)	(*n* = 72)	(*n* = 237)
MBL ± ESBL ± AmpC	16	53	90	16	52	90
OXA-48-like ± ESBL ± AmpC	8	21		3	14	
KPC ± ESBL		7				
GES carbapenemase ± ESBL		1	29		1	28
GES with undefined spectrum			1			1
AmpC ± ESBL	23					
ESBL only	187	60	22	4	2	22
None detected^b^	9	2	21		3	71
Not characterized	8	1	18			25

AmpC, Ambler class C β-lactamase; GES, Guiana ESBL; KPC, *Klebsiella pneumoniae* carbapenemase; OXA, oxacillinase.

^a^Intrinsic AmpC found in *P. aeruginosa* (PDC, *Pseudomonas*-derived cephalosporinase) is not shown in this analysis.

^b^No acquired β-lactamases included in the screening algorithm were detected.

## Discussion

Published studies describing *in vitro* surveillance data for ceftolozane/tazobactam tested against clinical isolates of Enterobacterales or *P. aeruginosa* from Mexico or countries in Latin America are very limited.^10^ The current study expands upon our previous publication describing the *in vitro* data for imipenem/relebactam tested against clinical isolates of Enterobacterales and *P. aeruginosa* from Latin American countries, including Mexico. The current study tests and describes a more current (2017–2021), larger isolate dataset from hospitalized patients in Mexico, includes testing of ceftolozane/tazobactam, and offers more in-depth analyses than our earlier publication.

We observed that ceftolozane/tazobactam inhibited 89% of *E. coli* and 87% of *K. pneumoniae* isolates (Table 1). An ESBL phenotype was frequently seen among both *E. coli* (64%) and *K. pneumoniae* (47%) in Mexico resulting in overall percent susceptible values of <50% for cefepime, ceftazidime and ceftriaxone. Previous studies of clinical isolates have also reported similar or even higher percentages of ESBL-positive *E. coli* and *K. pneumoniae* in Mexico.^17–19^ Ceftolozane/tazobactam was considerably more active than piperacillin/tazobactam (by 15 to 23 percentage points) against both *E. coli* (74% piperacillin/tazobactam-susceptible) and *K. pneumoniae* (64%) (Table 1). Ceftolozane/tazobactam percent susceptible values were also >18% higher for ESBL non-CRE phenotype *E. coli* (86% susceptible versus 68%) and *K. pneumoniae* (91% versus 48%) than values for piperacillin/tazobactam. A smaller study of 69 *E. coli* and 15 *K. pneumoniae* isolates collected in Mexico in 2016–2017 and tested against ceftolozane/tazobactam reported similar results to ours for all isolates tested and for isolates with an ESBL non-CRE phenotype.^10^ Cumulatively, these data argue strongly against the empirical use of piperacillin/tazobactam, cefepime, ceftazidime and ceftriaxone to treat a serious infection known or suspected to be caused by *E. coli*, *K. pneumoniae* or other less common Enterobacterales pathogens in Mexico without review of an isolate-specific antibiogram.

Seventy-seven percent (187/243) of ceftolozane/tazobactam-non-susceptible *E. coli* were shown to carry an ESBL as their only β-lactamase, 10% (24/243) carried an MBL or an OXA-48-like carbapenemase, 9% (23/243) carried AmpC (22 were CMY-type and one was MIR-type) with or without an ESBL, and 4% (9/243) of isolates did not have an acquired β-lactamase gene identified (Table 3). In contrast, the majority of ceftolozane/tazobactam-non-susceptible *K. pneumoniae* isolates (57%, 82/144) carried a carbapenemase gene (mostly an MBL or OXA-48-like carbapenemase). The 5%–6% difference observed between *E. coli* and *K. pneumoniae* in the percentages of isolates susceptible to imipenem/relebactam and the carbapenems (Table 1) is explained by a similar difference in the percentage of *E. coli* and *K. pneumoniae* isolates that carried an MBL or OXA-48-like carbapenemase gene (Table 2). Relebactam is inactive against Ambler class B (MBLs) and class D carbapenemases (e.g. OXA-48-like) and would not be expected to augment the activity of imipenem against isolates carrying these carbapenemases.^6^

Ceftolozane/tazobactam was the most active agent studied against *P. aeruginosa*, inhibiting 83% of isolates (Table 1). An earlier, smaller study of 127 *P. aeruginosa* isolates collected in Mexico in 2016–2017 reported a 19% lower percent susceptible value (64%) than our study; however, the isolates in the former study were also 17% to 22% less susceptible to meropenem (45%), piperacillin/tazobactam (50%) and ceftazidime (50%) than the isolates we tested and details of isolate collection and inclusion were not provided.^10^ In the current study, imipenem/relebactam (78% susceptible) was 5% less active than ceftolozane/tazobactam. However, a previous study reported that imipenem/relebactam remained active against a subset of ceftolozane/tazobactam-non-susceptible isolates, depending upon the β-lactamases and other resistance mechanisms present in the isolates, suggesting that both agents should be tested against clinical isolates, if possible.^20^ Specifically, the earlier study observed that in isolates of *P. aeruginosa* without a detected non-intrinsic β-lactamase gene, the presence of both a PDC (*Pseudomonas*-derived cephalosporinase) mutation and an indicator of PDC up-regulation frequently resulted in an imipenem/relebactam-susceptible/ceftolozane/tazobactam-non-susceptible phenotype.^20^ A second study confirmed the finding that ceftolozane/tazobactam-non-susceptible *P. aeruginosa* isolates without detected non-intrinsic β-lactamase genes were associated with mutations in PDC in combination with PDC up-regulation.^21^ Percent susceptible values for carbapenems, third- and fourth-generation cephems, piperacillin/tazobactam, and levofloxacin against *P. aeruginosa* were all compromised in the current study, ranging from 55% to 74% susceptible. Seventy-four percent (120/163) of characterized ceftolozane/tazobactam-non-susceptible *P. aeruginosa* isolates carried a carbapenemase gene and 13% carried only an ESBL; acquired β-lactamase genes were not detected in 13% of ceftolozane/tazobactam-non-susceptible *P. aeruginosa* isolates (Table 3).

In the current study, imipenem, meropenem and ertapenem percent susceptible values ranged from 97% to 99% for *E. coli* (>99% for ESBL non-CRE isolates) and from 91% to 93% for *K. pneumoniae* (>99% for ESBL non-CRE isolates) (Table 1). Earlier data for Enterobacterales isolates collected in Mexico from 2005 to 2015 showed comparable meropenem and imipenem percent susceptible values for *E. coli* (95% to >99%) but higher percent susceptible values for *K. pneumoniae* (95%–99%).^19,22,23^ In one report, 33% of Enterobacterales isolates from Mexico in 2012–2015 (*n *= 1862) were ESBL-positive and only 0.8% of isolates carried a carbapenemase.^22^ We estimated that 1.3% of *E. coli*, 7.5% of *K. pneumoniae* and 13.0% of *P. aeruginosa* isolates infecting hospitalized patients in Mexico in 2017–2021 carried a carbapenemase (Table 2), suggesting an increase in carbapenemase carriage among Enterobacterales over time. MBLs were the most prevalent carbapenemase type observed among *E. coli* (0.7% of all *E. coli* isolates), *K. pneumoniae* (4.8%) and *P. aeruginosa* (10.0%). In *P. aeruginosa*, the rate in the current study (10.0%) is double that reported in an earlier study from 2015 to 2017 (4.8% MBL among 524 isolates) from Mexico.^24^ Imipenem/relebactam percent susceptible values for *E. coli* and *K. pneumoniae* correlated closely with the presence of MBLs and OXA-48-like carbapenemases in the current study.

Seventy-eight percent of *P. aeruginosa* isolates in the current study were imipenem/relebactam-susceptible compared with percent susceptible values of 55% for imipenem and 62% for meropenem (Table 1). A previous report of 1794 *P. aeruginosa* isolates collected in hospitals in Mexico in 2012–2015 similarly reported that 65% of isolates were meropenem-susceptible.^22^ Fifty-six percent of characterized imipenem/relebactam-non-susceptible *P. aeruginosa* in the current study carried an MBL or GES carbapenemase; ESBLs were only identified in 10% of isolates and no acquired β-lactamase was detected in 33% of isolates (Table 3). In the isolates with ESBLs only or where no acquired β-lactamase gene was detected, OprD loss/mutation in combination with PDC derepression likely contributed to the imipenem/relebactam-non-susceptible phenotypes.^25^ Imipenem is known to be a strong inducer of PDC, and PDC expression levels have been correlated to imipenem MICs in *P. aeruginosa* with defects in OprD.^26^ Imipenem/relebactam activity against *P. aeruginosa* is unaffected by efflux-based resistance and is less affected by OprD loss than imipenem alone.^6^ Imipenem/relebactam also retains *in vitro* activity against isolates with KPC or PDC mutations that result in resistance to ceftazidime/avibactam or ceftolozane/tazobactam.^27^

Amikacin inhibited 91% of *E. coli*, 90% of *K. pneumoniae*, and 80% of *P. aeruginosa* using EUCAST breakpoints (Table 1). Even though it appears highly active *in vitro*, use of amikacin and other aminoglycosides is widely discouraged in clinical guidelines and by CLSI and EUCAST laboratory *in vitro* testing standards as all are associated with major toxicity and therapeutic limitations.^12,13,28^

The strengths of the current study are that it collected isolates from the same six sites in Mexico according to a consistent protocol for 5 years and used reference broth microdilution antimicrobial susceptibility testing and molecular testing performed in a central laboratory. Study limitations include that the number of medical centres participating was limited; sample quotas were used to collect isolates from different infection types that may affect the overall estimates of resistance and β-lactamase prevalence; isolates susceptible to ceftolozane/tazobactam and imipenem/relebactam were not molecularly characterized; and an ESBL non-CRE phenotype was defined as an isolate of *E. coli* and *K. pneumoniae* testing as non-susceptible to ceftriaxone (MIC ≥2 mg/L) and susceptible to ertapenem (MIC ≤0.5 mg/L) and did not include clavulanic acid-based phenotypic confirmatory testing.^12^

Based on our *in vitro* data, we conclude that ceftolozane/tazobactam and imipenem/relebactam may provide important treatment options for many patients infected with β-lactam-non-susceptible Gram-negative bacilli, including ESBL non-CRE *E. coli* and *K. pneumoniae* and β-lactam-non-susceptible *P. aeruginosa*. Increases in the prevalence of MBL-positive isolates of Gram-negative bacilli have been documented in Mexico and in Latin America and will erode the activities of all newer β-lactam/β-lactamase inhibitor combinations, including ceftolozane/tazobactam and imipenem/relebactam. Ongoing surveillance of the *in vitro* activities of established and newer antimicrobial agents against Gram-negative pathogens and monitoring for the spread of β-lactamase genes, particularly for MBLs, is critical.

## Supplementary Material

dlae077_Supplementary_Data

## Supplementary data

Tables S1 and S2 are available as Supplementary data at *JAC-AMR* Online.
