# Supplementary material for: Activity of ceftolozane/tazobactam and imipenem/relebactam against Gram-negative clinical isolates collected in Mexico—SMART 2017–2021
Source: JAC Antimicrob Resist. 2024 May 24;6(3):dlae077. doi: 10.1093/jacamr/dlae077 (PMC11126326; doi:10.1093/jacamr/dlae077)
Supplement: dlae077_Supplementary_Data [file dlae077_supplementary_data.docx]

**Supplementary Materials**

**Table S1.** Species distribution of all Gram-negative isolates collected by the SMART global surveillance program in Mexico from 2017 to 2021 stratified by infection source.

|  | Number of isolates | | | | | |
| --- | --- | --- | --- | --- | --- | --- |
| Species | BSI | IAI | LRTI | UTI | Not specified | Total |
| *Escherichia coli* | 408 | 782 | 315 | 829 | 3 | 2337 |
| *Klebsiella pneumoniae* | 224 | 189 | 420 | 293 | 1 | 1127 |
| *Pseudomonas aeruginosa* | 136 | 188 | 551 | 189 | 4 | 1068 |
| *Acinetobacter baumannii* | 82 | 50 | 342 | 44 | 2 | 520 |
| *Enterobacter cloacae* | 33 | 53 | 108 | 53 | 0 | 247 |
| *Klebsiella oxytoca* | 20 | 42 | 90 | 25 | 0 | 177 |
| *Stenotrophomonas maltophilia* | 11 | 10 | 139 | 7 | 2 | 169 |
| *Proteus mirabilis* | 14 | 29 | 28 | 79 | 1 | 151 |
| *Serratia marcescens* | 24 | 12 | 74 | 20 | 0 | 130 |
| *Morganella morganii* | 8 | 23 | 16 | 52 | 0 | 99 |
| Other species^a^ | 138 | 129 | 355 | 155 | 4 | 781 |
| Total | 1098 | 1507 | 2438 | 1746 | 17 | 6806 |

Abbreviations: BSI, bloodstream infection; IAI, intraabdominal infection; LRTI, lower respiratory tract infection; UTI, urinary tract infection.

^a^ The 781 Other species were comprised of 763 isolates of Other Enterobacterales species and 18 isolates of Other non-Enterobacterales species.

**Table S2.** Antimicrobial susceptibility of clinical isolates of *E. coli, K. pneumoniae,* and *P. aeruginosa* collected by the SMART global surveillance program in Mexico from 2017 to 2021 stratified by infection source

| Species |  | % Susceptible | | | | | | | | | | |
| --- | --- | --- | --- | --- | --- | --- | --- | --- | --- | --- | --- | --- |
| Infection source | n | C/T | IMI/REL | IMI | MEM | ETP | FEP | CAZ | CRO | P/T | LVX^a^ | AMK^b^ |
| *E. coli* |  |  |  |  |  |  |  |  |  |  |  |  |
| BSI | 408 | 90.4 | 98.5 | 97.5 | 98.0 | 95.6 | 37.3 | 40.7 | 31.6 | 75.2 | 28.7 | 91.4 |
| IAI | 782 | 89.6 | 99.5 | 99.0 | 99.4 | 98.0 | 37.1 | 43.4 | 32.4 | 76.6 | 32.3 | 91.4 |
| LRTI | 315 | 85.4 | 98.1 | 97.8 | 98.1 | 97.1 | 34.3 | 36.8 | 28.3 | 69.5 | 28.1 | 90.5 |
| UTI | 829 | 89.9 | 99.2 | 98.7 | 98.9 | 96.7 | 42.0 | 44.8 | 36.6 | 72.9 | 29.7 | 90.1 |
| *K. pneumoniae* |  |  |  |  |  |  |  |  |  |  |  |  |
| BSI | 224 | 82.6 | 92.0 | 89.7 | 90.2 | 88.8 | 35.3 | 35.7 | 29.9 | 59.8 | 49.1 | 83.0 |
| IAI | 189 | 91.0 | 95.2 | 93.7 | 93.7 | 92.1 | 49.7 | 50.8 | 45.0 | 61.9 | 52.8 | 93.1 |
| LRTI | 420 | 87.4 | 93.8 | 92.1 | 92.9 | 91.0 | 54.3 | 55.0 | 51.9 | 67.9 | 63.6 | 92.4 |
| UTI | 293 | 87.7 | 93.5 | 93.2 | 93.2 | 90.4 | 45.7 | 47.1 | 43.7 | 62.8 | 49.6 | 91.5 |
| *P. aeruginosa* |  |  |  |  |  |  |  |  |  |  |  |  |
| BSI | 136 | 75.7 | 73.5 | 54.4 | 61.8 | NA | 70.6 | 67.6 | NA | 69.9 | 68.4 | 76.5 |
| IAI | 188 | 83.0 | 81.4 | 58.0 | 61.7 | NA | 73.9 | 71.3 | NA | 68.6 | 66.0 | 80.3 |
| LRTI | 551 | 88.7 | 81.7 | 55.5 | 64.1 | NA | 78.0 | 77.0 | NA | 71.3 | 68.8 | 84.2 |
| UTI | 189 | 71.4 | 65.6 | 51.9 | 57.1 | NA | 63.5 | 60.3 | NA | 61.4 | 52.4 | 70.4 |

Abbreviations: C/T, ceftolozane/tazobactam; IMI/REL, imipenem/relebactam; IMI, imipenem; MEM, meropenem; ETP, ertapenem; FEP, cefepime; CAZ, ceftazidime; CRO, ceftriaxone; P/T, piperacillin/tazobactam; LVX, levofloxacin; AMK, amikacin; BSI, bloodstream infection; IAI, intraabdominal infection; LRTI, lower respiratory tract infection; UTI, urinary tract infection; NA, not applicable.

^a^ Levofloxacin was only tested against Enterobacterales in 2018-2021.

^b^ For *E. coli* and *K. pneumoniae*, the EUCAST breakpoint for amikacin was used because the concentration range tested did not extend low enough to include the revised 2023 CLSI susceptible breakpoint for that agent.
